# Hemodynamic and autonomic dysfunction in symptomatic carotid artery stenosis

**DOI:** 10.1111/cpf.70029

**Published:** 2025-09-21

**Authors:** Kristine Wichmann Madsen, Rasmus Primholdt Haahr, Tatevik Mkhitarjan, Niels Wiinberg, Jacob Rørbech Marstrand, Sverre Rosenbaum, Alexander Cuculiza Henriksen, Lisbeth Marner

**Affiliations:** ^1^ Department of Neurology Copenhagen University Hospital Bispebjerg Frederiksberg Copenhagen Denmark; ^2^ Department of Clinical Physiology and Nuclear Medicine Copenhagen University Hospital Bispebjerg Frederiksberg Copenhagen Denmark; ^3^ Department of Clinical Medicine University of Copenhagen Copenhagen Denmark; ^4^ Department of Nuclear Medicine Copenhagen University Hospital Herlev Copenhagen Denmark

**Keywords:** autonomic system, brain, cerebrovascular disease, stroke

## Abstract

**Background:**

Hemodynamic failure in patients with steno‐occlusive arterial disease is a major risk factor for stroke. Previous studies have identified impaired autonomic function in patients with carotid artery stenosis. Our study explores autonomic dysfunction and altered cerebrovascular hemodynamics in patients with stenosis and suspected hemodynamic failure.

**Methods:**

To assess autonomic nervous system dysfunction, patients underwent heart rate variability (HRV) testing, an active stand test, and the Valsalva maneuver with simultaneous monitoring of heart rate, blood pressure, and cardiac output. Transcranial Doppler was used to measure relative changes in cerebral blood flow during Valsalva.

**Results:**

Analysis of 13 patients and 19 controls revealed a significantly greater decrease in cerebral blood flow in the patient group during Valsalva, as evidenced by mean relative changes in time‐averaged peak velocities ±SE of 0.80 ± 0.04 in patients compared to 0.96 ± 0.05 in controls (*p* < 0.05). There were no significant differences in mean arterial blood pressure or heart rate during the Valsalva maneuver. HRV analysis and the active stand test did not reveal autonomic dysfunction or orthostatic intolerance.

**Conclusion:**

Patients with steno‐occlusive carotid artery disease exhibit impaired intracranial flow during Valsalva‐induced blood pressure reduction. However, our results do not support the presence of significant autonomic dysfunction in patients with symptomatic large‐vessel cerebrovascular disease as measured by HRV and blood pressure reduction during active stand.

## 1 INTRODUCTION

Ischaemic stroke is a leading cause of morbidity and mortality worldwide (Tsao et al., 2022). It is often caused by arteriosclerosis of the cervicocerebral arteries due to risk factors such as age, hypertension, diabetes, smoking, and hypercholesterolaemia (Kolominsky‐Rabas et al., 2001; Petty et al., 1999).

The brain, being among the most highly perfused organs, intricately regulates cerebral blood flow (CBF) to sustain its high metabolic demands. The healthy brain relies on cerebral autoregulation, which helps to maintain stable CBF during blood pressure fluctuations. This autoregulation is achieved by modulating intracranial vascular resistance (ICR) through changes in the diameter of the blood vessels (Lassen, 1959). However, in patients with severe steno‐occlusive arterial disease, cerebral autoregulation may become exceeded, leading to cerebral hypoperfusion (Henriksen et al., 2024; White and Markus, 1997), a condition referred to as hemodynamic failure. Patients with hemodynamic failure clinically present with ischaemic symptoms such as transient ischaemic attacks (TIAs), with limb‐shaking TIA being particularly indicative of hemodynamic failure (Henriksen et al., 2020; Knoflach et al., 2011).

Hemodynamic failure in patients with steno‐occlusive arterial disease is one of the greatest risk factors for stroke (Grubb, Jr, 1998), contributing to an estimated increased stroke risk of 15%–20% annually (Flaherty et al., 2004; Grubb et al., 2016; Powers, 2003). However, the pathophysiology remains largely unknown (Henriksen et al., 2020). One potential mechanism involves autonomic dysfunction, where the sympathetic/parasympathetic balance essential for regulating bodily functions, including heart rate (HR), blood pressure, and vascular tone, is disrupted.

Previous studies indicate that patients with carotid artery stenosis show signs of impaired autonomic function (Damkjær et al., 2023; Gautier et al., 2007; Gottsäter et al., 2006; Kwon et al., 2008; Xiong et al., 2012). Studies conducted by Gautier et al. (2007) and Gottsäter et al. (2006) have demonstrated that carotid atherosclerotic disease is associated with reduced heart rate variability (HRV). Similarly, Xiong et al. (2012), Kwon et al. (2008), and Damkjær et al. (2023) investigated post‐ischaemic stroke patients and found that those with carotid stenosis exhibited significantly greater impairments in autonomic function, including both parasympathetic and sympathetic measures, compared to patients without carotid stenosis. Other studies have demonstrated a significant effect of autonomic dysfunction on impaired cerebral autoregulation (Castro et al., 2014), further associating autonomic dysregulation with risks of cerebral hypoperfusion. Thus, autonomic dysfunction emerges as a plausible contributor to the impaired cerebrovascular autoregulation among patients with hemodynamic stroke or TIA. Nonetheless, a direct comparison between autonomic function and intracerebral hemodynamics has not been investigated.

This study investigates the dysregulation of CBF in patients with large vessel arteriosclerotic disease. We aim to discern whether reduced CBF is due to global autonomic dysfunction or localized to the area of stenosis‐induced flow limitation. To measure CBF, we utilize Transcranial Doppler ultrasound (TCD), a widely used noninvasive measurement of cerebral blood flow velocities and a reliable method for assessing cerebral perfusion and autoregulatory function (Bellapart and Fraser, 2009; Panerai, 2009). Several methods have been developed to assess cerebrovascular function using TCD, such as breath holding and acetazolamide tests (Dahl et al., 1995; Markwalder et al., 1984; Müller et al., 1995). These methods examine cerebrovascular responses under hyperaemic conditions, where blood flow increases following vasodilatory stimuli or metabolic challenges. In contrast, our study focuses specifically on hypoperfusion induced by the Valsalva maneuver, to mimic the pathophysiology in these patients.

The Valsalva maneuver is a validated method for assessing autonomic function, which challenges the cardiovascular and cerebrovascular systems, thereby revealing potential dysregulation not apparent under resting conditions. During the Valsalva maneuver thoracic pressure increases, thereby increasing the intracranial pressure (ICP) and reducing venous return, which temporarily lowers cerebral blood flow, allowing us to observe the cerebrovascular response to hypoperfusion. This is important, as impaired autoregulation in hypoperfused states may differ significantly from the hyperaemic responses typically measured in conventional tests, thereby offering a more precise understanding of hemodynamic compromise in high‐risk stroke patients. Integrating TCD with the Valsalva maneuver and simultaneous monitoring HR, blood pressure, and cardiac output (CO) provides valuable insights into cerebral autoregulation (Tiecks et al., 1996). We utilize further autonomic testing, such as HRV and active stand testing, to explore the parasympathetic/sympathetic balance and the presence of orthostatic intolerance as classical signs of autonomic dysfunction. Our study aimed to elucidate whether autonomic dysfunction contributes to the pathophysiology of hemodynamic failure.

## 2 METHODS

This study was conducted at the Department of Clinical Physiology and Nuclear Medicine, Copenhagen University Hospital Bispebjerg, Denmark. The study was approved by the Research Ethics Committee of the Capital Region of Denmark (H‐21049132), and written consent to participate was obtained from all individuals after receiving oral and written information according to the Helsinki Declaration. Data was handled according to the regulations by the Danish Data Protection Agency.

### 2.1 Study population

We recruited patients with ischaemic stroke or TIA, suspected to be caused by hemodynamic failure, from the Department of Neurology at Copenhagen University Hospital Bispebjerg. The inclusion criteria were occlusion or stenosis (>70%) of carotic artery or medial cerebral artery combined with symptoms of TIA. Exclusion criteria were pregnancy, reduced cooperability, previous extracranial‐intracranial bypass or stent operation. This expert evaluation was conducted by a senior vascular neurologist (SR), who carefully reviewed each patient's clinical history, clinical presentation, and relevant imaging studies to assess signs of hemodynamic compromise.

We assessed the patient's medication history to ensure that prescribed medicine did not unduly influence the autonomic nervous system and hemodynamic factors. Particular attention was given to medications such as alpha‐blockers, beta‐blockers, nitroglycerin, and statins.

We recruited a healthy age‐matched control group with no medical history of diabetes, previous cerebrovascular events, ischaemic heart or vascular disease, atrial fibrillation, or diagnosis of autonomic dysfunction.

### 2.2 Experimental protocol

Participants refrained from nicotine and alcohol for at least 12 h before evaluations and continued their usual medications. HR was monitored using an electrocardiogram (ECG), and arterial blood pressure was continuously measured using a noninvasive finger‐cuff device (Finapres Finometer Pro, Finapres Medical Systems B.V., Amsterdam, Netherlands). Data from the Finapres was recorded using LabChart 8.0 Software.

### 2.3 Heart rate variability (HRV) testing

Short‐term (5‐min) HRV recordings were conducted in the supine position after a 5‐min resting period. Recordings were made during spontaneous breathing using the Kubios HRV Standard software 3.5.0 (Kubios, Kuopio, Finland) and encompassed both time‐domain and frequency‐domain assessments.

### 2.4 Active stand test

Following a 5‐min resting period, active stand testing was performed (Finucane et al., 2019). Participants rose from a supine position and remained standing for 3 min, during which HR and blood pressure were continuously monitored. The obtained results included baseline measurements, the maximum and minimum blood pressure values with their respective times, and readings taken 3 min post‐standing.

### 2.5 Cerebrovascular hemodynamics during the Valsalva maneuver

We measured the relative change of the Time‐Averaged Peak Velocity (TAPV) in the M1 segment of the middle cerebral artery (MCA). Measurements were conducted using a GE LOGIQ E9 (GE Healthcare). In patients with intracranial stenosis, measurements were done after the jet. We assumed that a relative change in TAPV would reflect a similar change in the cerebral blood flow, i.e., assuming no major changes in the MCA diameter (Giller et al., 1993).

For patients whose MCA could not be identified due to a lack of a transtemporal window, a peripheral venous catheter was placed in the elbow for the administration of the contrast agent Sonovue® (5 mL of 8 μL/mL). In those cases, 2.5 mL was administered as a bolus before each of the two Valsalva maneuvers.

Valsalva maneuver was performed in a seated position with 20 s of forced expiration against a mouthpiece‐connected manometer, ensuring a targeted pressure of approximately 40 mmHg. Simultaneously, continuous TAPV, HR, mean arterial blood pressure (MAP), and cardiac output (CO) were recorded.

Recordings were systematically obtained at baseline and during the Valsalva maneuver at the following predefined time points: 5 s, 10 s, 15 s, and 20 s, during the time points with the lowest and highest TAPV and 15 s post‐Valsalva.

In the patient group, the Valsalva maneuver and the TCD measurements were conducted bilaterally, with a 5‐min resting period between each maneuver. Each vessel was preclassified as either symptomatic or asymptomatic based on the clinical information. Cerebrovascular hemodynamic variables were normalized to baseline values. The subsequent analysis compared changes in cerebrovascular hemodynamics by examining data from symptomatic versus asymptomatic hemispheres within the patient group, classified based on significant stenosis in either the MCA or internal carotid artery (ICA) on the respective side, and these results were further contrasted with data from the control group. In the healthy subjects, the Valsalva maneuver was only conducted in one hemisphere.

For patients, HR and MAP values for each time point were averaged between first and second Valsalva for subsequent comparison.

### 2.6 Calculating hemodynamic parameters

Vascular resistance can be estimated based on a modified version of Hagen–Poiseuille's law:

(1) $$\mathrm{Resistance}\;(R)=\frac{\mathrm{Pressure}\;\mathrm{gradient}\;(\Delta P)}{\mathrm{Flow}\;(Q)}.$$

Thus, total peripheral resistance (TPR) can be estimated from the pressure gradient (Δ*P*) divided by cardiac output (CO). We defined Δ*P* as the difference between MAP and the intrathoracic pressure (*V*_*p*_):

(2) $$\mathrm{TPR}=\frac{\mathrm{MAP}-V_{p}}{\mathrm{CO}}.$$

During rest, Vp was set to 0 mmHg, and during the Valsalva maneuver, it was set to 40 mmHg. To estimate ICR, we assumed that ICP would increase in parallel with the intrathoracic pressure due to venous congestion. Although ICP is likely slightly lower than intrathoracic pressure due to the head′s higher position, this difference should not influence our estimate of the change in pressure because blood pressure measurements were taken at heart level. Consequently, we assume that the MAP we use to calculate ΔP will have decreased proportionally to the flow measurements in the MCA. Please note that Δ*P* in the brain involves the same parameter referred to as the cerebral perfusion pressure (Silverman and Petersen, 2024).

(3) $$\mathrm{ICR}=\frac{\mathrm{MAP}-V_{p}}{\mathrm{TAPV}}.$$

### 2.7 Statistical analysis

Using Fisher's exact test, we compared baseline characteristics for categorical variables, expressed as frequencies and percentages. Continuous variables were analyzed using Welch Two‐Sample *t*‐test or Wilcoxon exact test, depending on whether data were normally distributed. We expressed continuous variables that followed a normal distribution as mean ± standard deviation (SD) or error (SE) in case of multiple time points, while non‐normally distributed continuous variables were reported as median and interquartile range (IQR).

We normalized continuous variables obtained during the Valsalva maneuver, such as TAPV, HR, MAP, and ICR, by calculating the ratio of each variable to its baseline value (e.g., TAPV_5sec_/TAPV_baseline_) to reflect relative changes from baseline. To compare relative changes in TAPV, HR, MAP, and ICR between groups, we first applied the Welch Two‐Sample *t*‐test. To correct for multiple measurements, we used a mixed‐effects linear regression model in which time points and groups were included as fixed effects, while individual subjects were treated as random effects. Relative values of TAPV at 15 s, 20 s, and max TAPV were further analyzed using receiver operating characteristic (ROC) curves to assess the diagnostic potential, with the optimal cutoff identified through Youden′s J‐index.

All statistical analyses were performed using the R statistical software package, version 4.3.0 (R Core Team, 2017; R Foundation for Statistical Computing, Vienna, Austria; https://www.R-project.org). *p* < 0.05 was considered statistically significant.

## 3 RESULTS

We included 13 patients (mean age 71.9 years ± 1.8; 4 females) and 19 elderly controls (mean age 67.4 ± 2.1 years, 3 females) in the study. Baseline characteristics of the participants are shown in Table 1. Baseline characteristics were generally comparable between control and patient groups, except for a higher frequency of statin use in the patient cohort (*p* < 0.001) and a significantly greater proportion of current or former smokers in the patient group compared to controls (*p* = 0.015). In the patient population, nine patients had stenosis in the right ICA, four patients in the left ICA, and one patient in the right MCA. Additionally, one patient had stenosis in the left anterior cerebral artery, and this hemisphere was therefore classified as asymptomatic.

**Table 1:** Baseline characteristics.

Characteristics	Control group, *N* = 19	Patient group, *N* = 13	*p* value
Age, years	67.4 ± 2.1‡	71.9 ± 1.8‡	0.11
Body Mass Index, kg/m^2^	25.6 (24.5, 29.5)†	25.5 (22.8, 27.5)†	0.60
Female sex, *n* (%)	3 (16%)	4 (31%)	0.40
Heart Rate, beats per minute	70.8 ± 9.6‡	72.3 ± 12.6‡	0.73
Mean Arterial Blood Pressure, mmHg	100.4 ± 15.0‡	101.4 ± 24.4‡	0.90
Cardiac output, L/min	6.7 ± 2.7‡	5.9 ± 1.7‡	0.35
Concomitant medication, n (%)			
Alpha‐blocker	0 (0%)	0 (0%)	
Beta‐blocker	0 (0%)	1 (7.7%)	0.40
Nitroglycerin	0 (0%)	0 (0%)	
Statin	5 (25%)	13 (100%)	**<0.001**
Comorbidities (*n*, %)			
Prior myocardial infarction	0 (0%)	1 (7.7%)	0.40
Diabetes mellitus	0 (0%)	2 (15%)	0.20
Hypertension	8 (42%)	7 (54%)	0.70
Heart failure	0 (0%)	2 (15%)	0.20
Smoking status (*n*, %)			**0.015**
Nonsmoker	14 (74%)	3 (23%)	
Current smoker	1 (5.3%)	2 (15%)	
Prior smoker	4 (21%)	8 (62%)	

*Note*: 1, Due to two patients with bilateral stenosis, *n* = 15. Bold values indicate statistically significant.

^†^ Median (IQR).

^‡^ Mean ± SD.

### 3.1 Heart rate variability

Valid ECG recordings were obtained in 12/13 patients and 17/19 controls. ECG data were excluded in one case due to frequent ventricular extrasystoles and in two cases due to technical errors. As seen in Table 2, HRV indexes were similar across groups with no evidence of autonomic dysfunction (Nunan et al., 2010).

**Table 2:** Heart rate variability analysis and active stand test.

Short‐term heart rate variability	Control group, *N* = 17†	Patient group, *N* = 12†	*p* value
Mean RR (ms)	869 (798, 952)	807 (695, 910)	0.3
SDNN (ms)	20 (16, 37)	20 (16, 29)	0.8
RMSSD (ms)	19 (12, 42)	19 (12, 31)	0.9
LF (ms^2^)	236 (146, 417)	203 (62, 394)	0.4
HF (ms^2^)	101 (56, 208)	74 (41, 133)	0.3
Total power (ms^2^)	362 (248, 635)	359 (110, 636)	0.4
LF/HF ratio	1.97 (1.17, 3.49)	2.24 (1.27, 3.58)	0.9

Active stand test	Control group, *N* = 20	Patient group, *N* = 13	*p*‐Value
drop in sBP > 20 mmHG, *n* (%)	0 (%)	0 (%)	
Max HR – Min HR (BPM)	18 (12, 22)†	13 (10, 14)†	0.07
Max MAP – Min MAP (mmHg)	45 (34, 50)†	30 (17, 45)†	0.06

*Note*: Mean RR: mean of time between two successive R‐waves (RR intervals); SDNN: standard deviation (SD) of time between normal heartbeats (NN interval); RMSSD: root mean square of successive RR Interval differences, measure of heart rate variability; LF Power: frequency activity of the low frequency band (0.04–0.15 Hz); HF Power: frequency activity of the high frequency band (0.15–0.4 Hz); LF/HF Ratio of Low Frequency‐to‐High Frequency power; sBP: systolic blood pressure, HR: heart rate; MAP: mean arterial pressure.

^†^ Median (IQR).

### 3.2 Active stand

The active stand test was completed for all participants. No participants developed orthostatic hypotension (Table 2), defined as a sustained blood pressure decrease of 20 mmHg systolic blood pressure (sBP) within 3 min of standing (Freeman et al., 2011), although one patient experienced mild dizziness during standing. The active stand test indicated a trend of diminished cardiovascular response in patients, with a smaller difference between maximum and minimum HR and MAP. In the patient group, the median increase in HR was 13 BPM (IQR: 10–14), compared to 18 BPM (IQR: 12–22) in the control group (*p* = 0.07). The median difference between minimum and maximum MAP was 30 mmHg (IQR: 17–45) for patients and 45 mmHg (IQR: 34–50) for controls (*p* = 0.06).

### 3.3 Valsalva maneuver and TCD assessment

**Figure 1:**
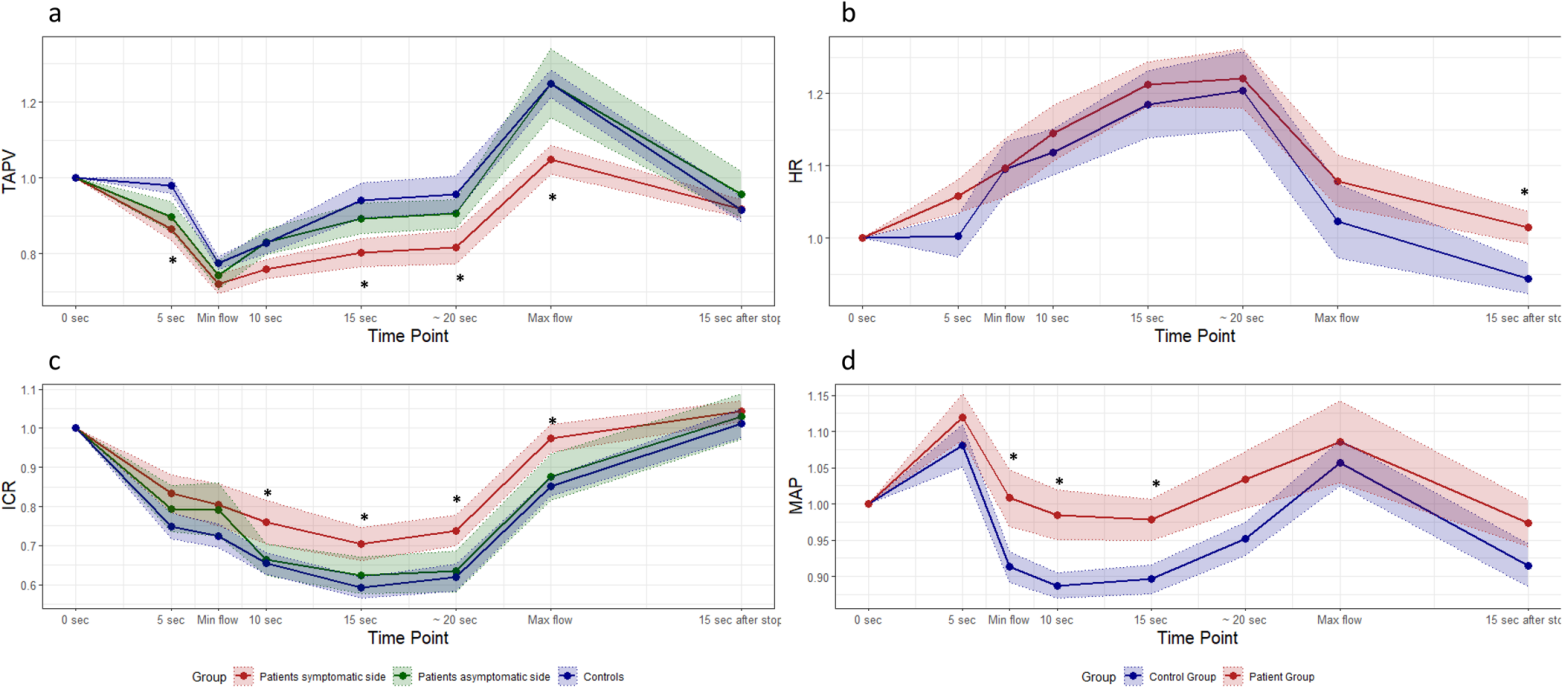
Values are shown relative to baseline (mean ± SE) for TAPV, HR, ICR, and MAP (a–d). *p < 0.05: patient group′s symptomatic side compared to the control group (a and b), and patient group compared to the control group (c, d). HR, heart rate; ICR, intracranial vascular resistance; MAP, mean arterial pressure; TAPV, Time‐Averaged Peak Velocity.

Relative changes in HR, MAP, TAPV, and ICR during the Valsalva maneuver are shown in Figure 1. TCD recordings were obtained in 13/13 patients and 18/19 controls. TCD measurements were excluded in one case, due to lack of a transtemporal window.

Analysis of TAPV and ICR showed that significant differences were present only between the patients' symptomatic hemisphere and the hemisphere of the healthy subjects, with no significant differences observed between the symptomatic and asymptomatic hemispheres within the patient group, nor between the asymptomatic hemisphere and the control hemisphere (Figure 1).

Using Welch Two Sample *t*‐test, we found that normalized TAPV (relative to baseline) was lower in the patient group′s symptomatic hemisphere compared to the control group at 5 s, 15 s, 20 s, and the time of max TAPV during the Valsalva maneuver (*p* < 0.05; Table 3). Furthermore, the normalized ICR in the symptomatic hemisphere of the patient group was significantly lower compared to the control group at 10 s, 15 s, 20 s and at the time of max TAPV (*p* < 0.05; Table 3). For HR, no significant differences in normalized values were observed between groups. However, the *normalized MAP* was significantly lower in the patient group at 10 s (*p* = 0.019) and 15 s (*p* = 0.035) compared to controls (Table 3).

**Table 3:** Cerebrovascular and hemodynamic changes during Valsalva maneuver.

Time point	Patient's symptomatic side, *N* = 13^1^	Patient's asymptomatic side, *N* = 13^1^	Control group, *N* = 18^1^
**Changes in TAPV relative to baseline**			
5 s	**0.87** ± **0.03** a	0.90 ± 0.04	0.98 ± 0.02
Time of min TAPV	0.72 ± 0.03	0.74 ± 0.03	0.78 ± 0.02
10 s	0.76 ± 0.03	0.83 ± 0.03	0.83 ± 0.03
15 s	**0.80 ± 0.04** a	0.89 ± 0.04	0.94 ± 0.05
20 s	**0.80 ± 0.04** a	0.91 ± 0.04	0.96 ± 0.05
Time of max TAPV	**1.05** ± **0.04** a	1.25 ± 0.09	1.25 ± 0.04
15 s after stop	0.92 ± 0.02	0.95 ± 0.06	0.92 ± 0.03
**Changes in ICR relative to baseline**			
5 s	0.86 ± 0.05	0.79 ± 0.06	0.75 ± 0.03
Time of min TAPV	0.85 ± 0.05	0.79 ± 0.07	0.73 ± 0.03
10 s	**0.78** ± **0.06** a	0.66 ± 0.04	0.65 ± 0.03
15 s	**0.73** ± **0.04** a	0.62 ± 0.05	0.59 ± 0.03
20 s	**0.76** ± **0.04** a	0.63 ± 0.05	0.62 ± 0.04
Time of max TAPV	**0.97** ± **0.04** a	0.88 ± 0.06	0.85 ± 0.02
15 s after stop	1.04 ± 0.03	1.03 ± 0.06	1.01 ± 0.04

	Patient group, *N* = 13^1^	Control group, *N* = 19^1^	*p* value
**Changes in MAP relative to baseline**			
5 s	1.10 ± 0.03	1.08 ± 0.03	0.372
Time of min TAPV	1.01 ± 0.04	0.91 ± 0.02	**0.045**
10 s	0.98 ± 0.03	0.89 ± 0.02	**0.019**
15 s	0.98 ± 0.03	0.90 ± 0.03	**0.035**
20 s	1.03 ± 0.04	0.95 ± 0.02	0.122
Time of max TAPV	1.09 ± 0.06	1.06 ± 0.03	0.696
15 s after stop	0.97 ± 0.03	0.92 ± 0.03	0.187
**Changes in HR relative to baseline**			
5 s	1.06 ± 0.02	1.00 ± 0.03	0.136
Time of min TAPV	1.10 ± 0.04	1.10 ± 0.04	0.983
10 s	1.14 ± 0.04	1.12 ± 0.03	0.613
15 s	1.21 ± 0.03	1.19 ± 0.05	0.634
20 s	1.22 ± 0.04	1.20 ± 0.05	0.813
Time of max TAPV	1.08 ± 0.04	1.02 ± 0.05	0.400
15 s after stop	1.01 ± 0.02	0.94 ± 0.02	**0.029**
Valsalva ratio (max HR/min HR)	1.32 ± 0.01	1.59 ± 0.02	<0.001

*Note*: Bold values indicate statistically significant.

Abbreviations: HR, heart rate; ICR, intracranial vascular resistance; TAPV, Time‐Averaged Peak Velocity.

^1^ Mean ± SE.

^a^ *p* < 0.05 when comparing the patient group′s symptomatic side with the control group using a *t*‐test.

A mixed‐effects linear regression model confirmed that significant differences between the symptomatic side of the patient and the control group were maintained for TAPV and ICR at 15 s, 20 s, and the time of max TAPV (*p* < 0.05). Like the unadjusted tests, the mixed‐effects model showed no statistically significant differences in relative HR between patients and controls. The mixed‐effects linear regression model shows a trend toward lower MAP was noted in the patient group compared to controls at the time of min TAPV (*p* = 0.070), 10 s (*p* = 0.054), and 15 s (*p* = 0.068).

Comparison of mean Valsalva ratio (max HR/min HR) using Welch Two Sample *t*‐test revealed significantly lower Valsalva ratio in patients (*p* < 0.001), with a mean Valsalva ratio of 1.32 ± 0.01 in the patient group and 1.59 ± 0.02 in the control group (Table 3).

During the Valsalva maneuver, one patient experienced limb‐shaking TIA, one patient experienced unilateral sensory disturbances in the upper extremity, and one patient became dizzy.

### 3.4 TCD and Valsalva maneuver as a diagnostic tool

**Figure 2:**
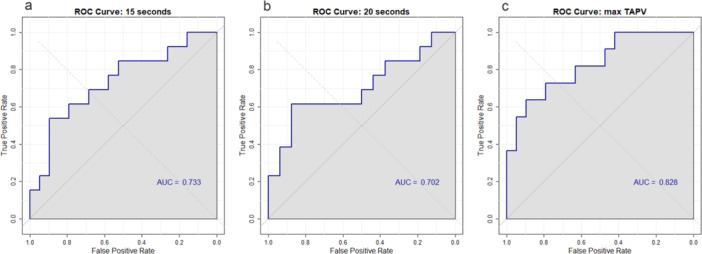
ROC curves showing the diagnostic performance of TAPV at 15 s (a), 20 s (b), and maximum flow (c). TAPV, Time‐Averaged Peak Velocity.

A ROC curve analysis was performed to assess the diagnostic ability of TAPV at 15 s, at 20 s (maximum flow), and at the point of maximum flow to distinguish flow in patients' symptomatic hemisphere from controls (Figure 2). At 15 s, the optimal TAPV cutoff for differentiating these groups was determined to be 0.79, with a sensitivity of 53.8% and a specificity of 89.5%. At 20 s, the cutoff was 0.80, with a sensitivity of 61.5% and a specificity of 87.5%. The maximum flow cutoff was calculated to be 1.07, yielding a sensitivity of 63.6% and a specificity of 89.5%.

## 4 DISCUSSION

Our study implies that patients suspected of hemodynamic failure due to steno‐occlusive arterial disease experience a reduction in CBF during Valsalva‐induced hypotension. Interestingly, this reduction was not attributable to a drop in MAP, but rather to a smaller decline in ICR.

This finding aligns with the expectation that patients with compromised large‐vessel circulation may have a reduced capacity to lower ICR, as this typically requires arterial vasodilation—a process likely already maximized in these patients due to reduced cerebral perfusion pressure due to vessel stenosis. This mechanism, central to cerebral autoregulation, is well understood: if cerebral perfusion pressure falls below a critical threshold, autoregulation can no longer sustain cerebral blood flow (Aaslid et al., 1989). This was also clinically evident in at least two patients with symptoms corresponding to a TIA during the Valsalva.

Our findings do not demonstrate the presence of global autonomic dysfunction in stroke patients with large‐vessel stenosis. Although HRV, active stand, and Valsalva maneuver assessments did not reveal significant differences between patients and controls, some observed trends suggest potential dysregulation in autonomic responses under physiological stress.

During the Valsalva maneuver, patients showed a slightly higher MAP (Figure 1d) and a lower Valsalva ratio (Table 3) in patients likely reflecting a compensatory response to maintain cerebral perfusion pressure. Likewise, in the active stand test, a trend toward reduced cardiovascular response was noted, with smaller changes in MAP (*p* = 0.06) and heart rate (*p* = 0.07) (Table 2), suggesting a blunted autonomic response to orthostatic challenge. However, these compensatory mechanisms do not suggest an autonomic dysfunction.

Previous studies have reported an increased prevalence of autonomic dysfunction in a broad population of patients with carotid artery stenosis, including patients with asymptomatic stenosis (Gautier et al., 2007; Gottsäter et al., 2006) and those experiencing acute ischaemic stroke (Damkjær et al., 2023; Kwon et al., 2008; Xiong et al., 2012). In contrast, our study focuses on a specific subpopulation with recurrent TIAs and demonstrable hemodynamic failure rather than patients in the acute phase of stroke. This focus may explain some of the discrepancy between our and previous results. Further, our study is strengthened by an age‐ and sex‐matched control group.

Although consistently lower/higher, we found no significant differences in TAPV and ICR between the symptomatic and asymptomatic sides. We had expected a side difference as this is the key finding in perfusion scans during vessel dilation tests. The lack of difference in this study is likely due to the limited number of included patients.

Following our findings on TAPV, we conducted a ROC curve analysis to differentiate affected hemispheres from those of healthy participants. This analysis yielded a moderate to strong classification with an AUC > 0.80. Although this analysis was exploratory and not part of our initial study plan, it provides an intriguing potential for developing a simple assessment of hemodynamic failure during hypotension. Such an approach might prove useful reflecting clinical conditions during ischaemic events (Henriksen et al., 2024).

The strength of the study is the inclusion of carefully selected patients with ongoing symptoms of hemodynamic failure. However, several limitations must be considered. The limited sample size increases the risk of type II errors and limits the statistical power to detect the effects of varying degrees of stenosis severity on cerebrovascular hemodynamics and systemic responses to the Valsalva maneuver. Additionally, hemodynamic failure is a challenging diagnosis with symptoms similar to those of embolic stroke that is often seen in patients with severe stenosis.

For cerebral blood flow assessments, we used TAPV measured by TCD, which assumes a stable MCA diameter—a factor we cannot control in this dynamic setting. While TCD is the current method of choice for measuring dynamic changes in cerebral perfusion during physiological stress, an alternative approach using HMPAO SPECT has been proposed for such assessments (Henriksen et al., 2024).

Another limitation is that control participants did not undergo comprehensive assessment of their cervicocerebral arteries, leaving uncertainty regarding asymptomatic carotid atherosclerosis. Given the age and sex composition of the control group, it is plausible that some participants may have undetected large‐vessel atherosclerotic disease; however, the overall prevalence of asymptomatic carotid stenosis is relatively low. Furthermore, the examiners were not blinded to the presence of symptomatic carotid stenosis, introducing a potential risk of interpretation bias. While the sex distribution between groups was not entirely balanced, we do not believe this has materially affected the study outcomes.

Evidence suggests that intake of statins may diminish the sympathetic activity and thereby alter the balance between the sympathetic and parasympathetic nerve systems (Lewandowski et al., 2015; Millar and Floras, 2014). However, all our patients had prescribed statins precluding evaluation in the present study.

Finally, although noninvasive measurements using Finapres are inherently limited, focusing on relative hemodynamic changes minimizes these constraints. Movement during the Valsalva maneuver could influence probe positioning and TCD measurements. However, in our experience, headband fixation is often more vulnerable to patient movement; hence, a free‐hand technique using triplex was adopted in this study.

## 5 CONCLUSION

Patients with presumed hemodynamic failure exhibit impaired intracranial flow during Valsalva‐induced blood pressure reduction. However, our results do not support autonomic dysfunction in patients with symptomatic large‐vessel cerebrovascular disease as measured by HRV and blood pressure reduction during active stand.

## AUTHOR CONTRIBUTIONS

Sverre Rosenbaum, Alexander Cuculiza Henriksen, Tatevik Mkhitarjan, and Lisbeth Marner conceived and planned the experiments. Tatevik Mkhitarjan, Rasmus Primholdt Haahr, Alexander Cuculiza Henriksen, Lisbeth Marner, and Niels Wiinberg carried out the pilot experiments. Sverre Rosenbaum and Jacob Rørbech Marstrand included the patients. Kristine Wichmann Madsen, Niels Wiinberg, and Alexander Cuculiza Henriksen planned and carried out the experiments. Kristine Wichmann Madsen and Alexander Cuculiza Henriksen analyzed the data. Kristine Wichmann Madsen, Niels Wiinberg, Jacob Rørbech Marstrand, Sverre Rosenbaum, Alexander Cuculiza Henriksen, and Lisbeth Marner contributed to the interpretation of the results. Kristine Wichmann Madsen and Alexander Cuculiza Henriksen took the lead in writing the manuscript. All authors provided critical feedback and helped shape the analysis and manuscript. All authors have approved the final version of the manuscript.

## CONFLICT OF INTEREST STATEMENT

The authors declare no conflicts of interest.

## Data Availability

The data included in the present study is available on reasonable request to the corresponding author. The data that support the findings of this study are not publicly available due to national and local legal requirements regarding privacy issues but are available from the corresponding author upon reasonable request.
